# Professional Quality and Empathy of Responses Provided by AI and Dentists: A Cross-Sectional Study

**DOI:** 10.3390/healthcare13233099

**Published:** 2025-11-27

**Authors:** Thekla J. Pfeiffer-Grötz, Luisa Schäfer, Anke Hollinderbäumer, Dominik Haag, Lisa Zöll, James Deschner

**Affiliations:** 1 Department of Periodontology and Operative Dentistry, University Medical Center of the Johannes Gutenberg University, 55131 Mainz, Germany; sluisa@students.uni-mainz.de (L.S.); dominik.haag@unimedizin-mainz.de (D.H.); lisa.zoell@unimedizin-mainz.de (L.Z.); james.deschner@unimedizin-mainz.de (J.D.); 2 Rudolf Frey Lernklinik, University Medical Center, University of Mainz, 55131 Mainz, Germany; hollinde@uni-mainz.de

**Keywords:** chatbot, ChatGPT, dentistry, patient communication, empathy, response quality, digitalization in healthcare, AI in medicine, patient satisfaction

## Abstract

**Background/Objectives:** Artificial intelligence–based language models such as ChatGPT are increasingly used in medical communication, yet their performance compared with human clinicians remains insufficiently explored in dentistry. Because communication quality, including accuracy and empathy, is essential for patient understanding, this study aimed to compare ChatGPT’s responses with those of dentists with different levels of professional experience. **Methods:** Ten standardized dental patient questions were generated by the authors and answered by ChatGPT and by three dentist groups (<2 years, 2–5 years, >5 years of experience; one respondent per group, randomly selected from five eligible dentists). Subsequently, 30 dentists rated the professional quality of the responses, and 50 patients evaluated perceived empathy on 4-point scales. Group differences were analyzed using the non-parametric Friedman test with exact post hoc comparisons and Bonferroni correction. **Results:** ChatGPT received higher ratings than all dentist groups in both domains. Mean empathy scores were 3.23 for ChatGPT versus 1.73–2.14 for dentists, and mean quality scores were 3.50 versus 1.79–2.21 (all *p* < 0.001). Early-career dentists scored moderately higher than the most experienced group but consistently below ChatGPT. Due to the exploratory design and small number of respondents per experience group, these findings should be interpreted cautiously. **Conclusions:** ChatGPT generated responses rated as more empathetic and of higher professional quality than those of participating dentists. This suggests potential value for supporting routine, text-based dental communication. However, limitations such as lack of genuine empathy, data privacy concerns, and clinical responsibility must be considered. Larger studies are needed to validate these results

## 1. Introduction

Digitalization has become increasingly important in almost all areas of life in recent years—including the healthcare sector. Digital processes are increasingly being introduced to make workflows more efficient and improve patient care. Despite this progress, the digitalization process remains incomplete in many medical areas. The COVID-19 pandemic acted as an additional catalyst, but the need for digital innovation already existed beforehand. The World Health Organization (WHO) was quick to emphasize the central role of digital technologies for a modern healthcare system and highlighted their potential to improve quality and accessibility [1].

One example of this development is the increased use of electronic patient records, which has increased significantly since 2020. At the same time, the increasing number of electronic patient messages has led to additional work for medical staff, which can lead to conflicts between administrative and patient-centered activities [2]. A future challenge will be to manage these digital requests efficiently without placing an additional burden on healthcare professionals.

The development of artificial intelligence (AI) is also progressing rapidly in parallel with the digital transformation. Having been researched for over five decades, AI systems now have concrete applications in everyday life and increasingly also in medicine [3]. The first areas of application are already being investigated in dentistry—for example in caries diagnostics [4], implant prognosis [5] or the classification of periodontally damaged teeth [6].

However, communication remains a central element of dental work. This is essential for education, participatory decision-making and building trust—especially when it comes to preventive measures against caries and periodontitis [7]. Dentistry is a particularly relevant field for the evaluation of empathy, as dental treatment is strongly associated with anxiety, invasiveness, and pain-related concerns, which require a high degree of supportive and patient-centered communication. Compared with many other specialties, dentists often operate in short but emotionally intense interactions, in which empathic verbal reassurance plays a decisive role in patient cooperation and satisfaction. With this in mind, this study examines the potential of AI-supported chatbots, specifically ChatGPT, in dental patient communication. Chatbots are one of the most widespread forms of human–computer interaction and can respond in a human-like manner using text or voice-based input [8].

The aim of this study was to compare the content quality and empathy of responses provided by ChatGPT and dentists with varying levels of professional experience to questions typically asked by patients to dentists. Opportunities, challenges and ethical-legal implications are discussed—especially with regard to data protection, responsibility and the relationship of trust in doctor-patient communication. Relevant aspects such as dealing with dental anxiety [9,10] and the potential relief of routine requests are also considered. The results of this work could contribute to the evidence-based integration of modern AI technology in dentistry.

Based on the existing literature, we hypothesized that ChatGPT responses would be perceived as more empathetic than those of dentists, while not necessarily outperforming dentists in technical accuracy.

## 2. Materials and Methods

### 2.1. Study Design

The present study was designed as a cross-sectional study with the aim of comparing the response performance of an AI-based chatbot (ChatGPT Version 3.5, OpenAI, San Francisco, CA, USA) with that of dentists. Ten standardized questions, which dentists typically are asked by patients, were generated by ChatGPT. The content of these questions was provided by the authors of this study. These questions were then answered by dentists with varying levels of professional experience and ChatGPT itself. Finally, another group of dentists evaluated the responses to these questions in terms of professional quality, while patients assessed the responses with regard to empathy. Both the dentists and patients ranked each of the answers to the 10 questions from 1 to 4.

### 2.2. Thematic Content of the Questions

Ten standardized questions related to practice-relevant topics and thereby often asked by patients in the dental office were generated by ChatGPT. The content of these questions was as follows: (a) caries prophylaxis in children, (b) causes and treatment of bad breath, (c) toothache with sensitivity to cold, (d) bleeding gums, (e) options for pain relief during root canal treatment, (f) effects of smoking on oral health, (g) influence of diabetes on the teeth, (h) fear of dental treatment, (i) fissure caries in adolescents, and (j) risks due to excessive use of mouthwash.

### 2.3. Response to the Questions by AI and Dentists

The ten questions were answered by three groups of dentists with varying lengths of professional experience (5 dentists per group) and by ChatGPT 3.5. Group 1 consisted of dentists with <2 years of professional experience, group 2 with 2–5 years of professional experience and group 3 with >5 years of professional experience. The AI application ChatGPT and dentists were asked to answer the ten questions as follows: “You are my dentist. Answer this question in four short sentences.”. To ensure transparency, professional experience was defined as the number of full years of practical clinical activity after obtaining the dental license (Approbation). The responses from one dentist from each experience group were selected at random, so that ultimately 10 responses from each of the three experience groups were available for further evaluation. As only a single dentist per experience category contributed the final set of responses, the design of the study inherently reflects an exploratory/pilot character, and the findings should be interpreted within this context. The experience intervals (<2, 2–5, >5 years) were chosen because they represent typical stages of early-career development within the participating university clinic. This wording was intended to promote both professional and patient-oriented communication through AI.

### 2.4. Evaluation of Answers by Patients and Dentists

The responses from ChatGPT and dentists with varying levels of professional experience were anonymized and then submitted to other dentists (n = 30) and patients (n = 50) for evaluation.

The dentists evaluated the responses to these questions in terms of their professional quality, while the patients assessed the responses in terms of empathy. The content of the evaluation was pointed out in each case. Patients received a standardized written definition of empathy to ensure a consistent understanding of the construct. Dentists, in contrast, were instructed to evaluate only the professional content quality and therefore did not receive an empathy definition. Both dentists and patients rated each of the answers to the 10 questions on a scale of 1 to 4. All evaluator groups were fully blinded to the origin of each response; specifically, they did not know whether an answer originated from a dentist or from ChatGPT, nor to which experience group the dentist belonged. The 30 participating dentists were recruited via voluntary participation from different departments of the university dental clinic. No additional inclusion or exclusion criteria were applied beyond professional qualification as licensed dentists. Patients were recruited in the waiting area of the university dental clinic and also participated voluntarily without further inclusion or exclusion criteria apart from being adult (≥18 years). For the generation of the dentist responses, each experience group originally consisted of five authorized dentists. One dentist per group was selected at random to provide the final set of responses. The evaluators were not informed about this procedure.

### 2.5. Statistical Analysis

The data analysis was carried out using the software R: A language and environment for statistical computing version 4.4.3 (R Foundation for Statistical Computing, Vienna, Austria). As the requirements for an analysis of variance (normal distribution and homogeneity of variance) were not met, non-parametric methods were used. To examine differences between groups in repeated measures, the Friedman test was used as a non-parametric alternative to ANOVA. We conducted pairwise post hoc comparisons following the Friedman test using the exact procedure proposed by Eisinga Et Al. Eisinga et al. (2017) [11]. The Bonferroni correction was used to correct the alpha error in multiple comparisons. Given the ten questionnaire items, this resulted in an adjusted significance level of *p* < 0.005. Correlation analyses were performed to examine possible correlations between the assessed dimensions of empathy and response quality. The results were also presented graphically using the software R: A language and environment for statistical computing version 4.4.3 (R Foundation for Statistical Computing, Vienna, Austria).

Given the exploratory nature of the study design and the small sample structure, inter-rater reliability measures (e.g., intraclass correlation coefficients) and calibration procedures between raters were not performed. This represents a methodological limitation and is addressed in the Discussion.

Although non-parametric data are commonly summarized using medians and interquartile ranges, means and standard deviations were additionally reported to allow comparability with existing literature and in line with recommendations from the statistical advisory unit.

The non-parametric Friedman test was used to investigate possible differences in the assessment of empathy between the four groups (dentists with less than two, two to five and more than five years of professional experience and ChatGPT). This is suitable for analyzing rank data in linked samples and was used here as a nonparametric alternative to repeated-measures ANOVA due to the data structure and the violation of assumptions.

Due to the limited sample size and the structure of the dataset, more advanced statistical approaches such as mixed-effects models were not applicable in the current study but are recommended for future research.

## 3. Results

### 3.1. Analysis of the Survey of Dentists

The survey included responses from a total of 30 dentists and was analyzed with regard to gender, age, and professional experience (Table 1).

The mean scores were calculated for each question and group (Table 2, Figure S1). For question 1, the mean difference between ChatGPT and dentists with less than two years of professional experience is 1.233 points (95% confidence interval: 0.94 to 1.53). Compared to the group with two to five years of experience, the difference is 1.6 points (95% CI: 1.31 to 1.89), and compared to the group with more than five years of experience, the difference is 2.9 points (95% CI: 2.61 to 3.19), which represents the largest difference (Table 3, Figure S2). A similar pattern can be seen in question 5, with differences of 0.767 points compared to the group with <2 years of experience (95% CI: 0.30 to 1.23), 1.7 points for the 2–5 years group (95% CI: 1.24 to 2.16) and 2.333 points for the >5 years group (95% CI: 1.87 to 2.80) (Table 3, Figure S2). This trend continues in questions 9 and 10 (Table 3, Figure S2). In most cases, the confidence intervals are completely above zero, indicating statistically significant differences. Questions 6 and 7 are exceptions: here, the confidence intervals overlap with zero, so no significant difference can be determined. In question 6, this applies to the comparison with the group >5 years (mean difference 0.600; 95% CI: −0.11 to 1.31). For question 7, this applies to the groups with 2–5 years (0.567; 95% CI: −0.12 to 1.25) and >5 years (0.233; 95% CI: −0.45 to 0.92) (Table 3, Figure S2).

Overall, the results show that ChatGPT was rated significantly better than dentists of all experience levels for most questions. The largest deviations are found in questions 1, 5, 8, 9, and 10, while no significant differences were found for questions 6 and 7 in relation to certain groups. An overview is presented in the following table.

### 3.2. Analysis of the Patient Survey

The evaluation was based on responses from a total of 50 patients. Gender and age distribution were analyzed (Table 4).

The Friedman test revealed significant differences between the four groups for all ten questions (*p* < 0.001). The chi-square values ranged from X^2^ = 31.11 to X^2^ = 73.65, indicating systematic differences in patient-perceived empathy. Since the Friedman test only identifies overall group differences, exact pairwise post hoc tests according to Eisinga et al. (2017) [11] were performed. These analyses showed that ChatGPT was rated significantly higher than all three dentist groups for the majority of items. In most comparisons, the 95% confidence intervals of the mean differences lay entirely above zero, confirming significantly higher empathy ratings for ChatGPT.

A summary of the mean empathy ratings across all 10 questions is presented in Table 5.

The mean values of the ratings for perceived empathy were calculated for each of the ten questions and for all four groups (dentists with <2, 2–5 and >5 years of professional experience and ChatGPT). The mean scores were calculated for each question and group (Table 6, Figure S3).

For question 1, the mean difference between ChatGPT and dentists with less than two years of professional experience was −0.02 points (95% confidence interval: −0.50 to 0.46), indicating no significant difference. A similar non-significant result was found for question 2 (−0.16; 95% CI: −0.68 to 0.36). Question 4 also showed no significant difference when comparing ChatGPT with dentists with 2–5 years of experience (0.00; 95% CI: −0.49 to 0.49). For question 7, no significant differences were observed in the 2–5 years group (0.42; 95% CI: −0.11 to 0.95) or the >5 years group (0.40; 95% CI: −0.13 to 0.93) (Table 7, Figure S4). By contrast, significant differences in favor of ChatGPT were observed for several questions. In question 6, ChatGPT outperformed dentists with more than five years of professional experience (2.16; 95% CI: 1.73 to 2.59). In question 9, ChatGPT was rated significantly higher than dentists with less than two years of experience (2.02; 95% CI: 1.56 to 2.48). In question 10, ChatGPT again showed a significant advantage over dentists with more than five years of experience (2.05; 95% CI: 1.61 to 2.49) (Table 7, Figure S4).

### 3.3. Correlation Between the Evaluation of Response Quality and Empathy

To test whether responses that were perceived as empathetic by patients were also rated positively in terms of quality, the mean values of both surveys were combined and evaluated using a correlation analysis. The Spearman correlation matrix yielded a correlation coefficient of 0.8097 between empathy and response quality, indicating a strong positive correlation (Figure S5).

The corresponding *p*-value < 0.001 also confirms the statistical significance of this correlation. To illustrate this correlation, the mean values of both assessments are shown in the figure below. The scatter plot below illustrates the positive correlation between the two variables.

## 4. Discussion

This study shows that the AI-based chatbot ChatGPT was rated significantly better in terms of response quality compared to the participating dentists. The group mean value of the chatbot was 3.50 and thus clearly exceeded the ratings of all dentist groups, especially those of dentists with more than five years of professional experience (Ø = 1.79). This difference was statistically significant and especially pronounced for specific questions, indicating that ChatGPT may play an increasingly important role in patient communication. However, this finding applies only to short, text-based standardized questions and should not be generalized to complex clinical scenarios or multimodal communication settings. These findings suggest that ChatGPT could serve as a valuable support tool in dental communication, particularly for preparing and following up on patient interactions.

These results are in line with studies by Yeo et al. (2023) and Ayers et al. (2023), in which ChatGPT was able to generate consistent, correct and high-quality answers to medical questions [12,13]. For example, ChatGPT showed high accuracy rates in gastroenterology consultations (cirrhosis: 79.1%; HCC: 74.0%). In another cross-sectional study, it was also found that ChatGPT was significantly more convincing with answers rated “good” or “very good” (78.5% vs. 22.1% for doctors). ChatGPT’s ability to draw on an extensive training corpus while not suffering from human fatigue or cognitive limitations is likely to have contributed significantly to its consistently high quality. The results of the study suggest that chatbots such as ChatGPT could be a useful addition, especially for answering standardized and frequently asked questions in dental consultations. At the same time, studies such as that by Munir et al. (2024) [14] show that specialists are critical of the performance of ChatGPT in more clinically complex contexts: Only 20% of the experts surveyed considered AI to be a reliable source of information in gastrointestinal surgery. This discrepancy emphasizes the need for a differentiated classification of the range of applications of AI in everyday medical practice. ChatGPT also performed significantly better than all participating dentists in the patient survey when it came to evaluating empathy. The chatbot achieved an average score of 3.23, while the dentist with more than five years of experience only scored 1.73. The difference was statistically significant and underlines ChatGPT’s ability to generate empathetic responses. One possible explanation lies in the targeted language training of modern language models, which aims to generate empathetic, well-formulated responses. However, this is simulated empathy, as the system lacks genuine emotional perception. The positive perception by patients could have been reinforced by the halo effect [15]: Well-structured and understandable answers may also lead to the assumption of increased empathy. Empathy is a central component of successful doctor-patient communication [16] and is closely linked to compliance, patient satisfaction and willingness to undergo treatment [17]. Studies show that a lack of empathy, inadequate information or ignoring patient concerns are often triggers for legal action [18]. It is particularly striking that the assessment of empathy, similar to the quality of response, decreases with professional experience. This result corresponds with research findings which show that a decline in empathic abilities can be observed as medical training and professional practice progresses [19,20]. Causes cited include emotional exhaustion, routine communication patterns and a lack of continuous further training. The correlation analysis revealed a strong positive correlation (r = 0.822; *p* < 0.001) between the assessment of response quality (by dentists) and perceived empathy (by patients). This indicates that both evaluation dimensions are closely linked, a well-structured and technically correct answer tends to be perceived as more empathetic. This interaction can also be explained by the halo effect mentioned above. The language, clarity and comprehensibility could be unconsciously interpreted as signs of empathic communication, even if there is no real interpersonal relationship. Despite the benefits, the use of AI also poses considerable challenges. Studies show that older people in particular, people without access to digital devices or with lower digital skills could be structurally disadvantaged [21]. A lack of trust, a lack of emotional connection and data protection concerns also represent hurdles. The acceptance of chatbots depends heavily on transparency, design and perceived control. There are also technical limitations: ChatGPT can generate incorrect information, reproduce stereotypical thought patterns or respond inconsistently [22]. This unreliability makes a critical examination of every response essential. Importantly, no linguistic bias is expected to have influenced the results, as all questions, responses, and evaluations were conducted exclusively in German. Therefore, differences in perceived empathy cannot be attributed to cross-language variation in tone or phrasing.

This study has several limitations. First, the overall sample size was small, and only one dentist per professional experience group contributed responses, which limits statistical power and generalizability. Second, no information was collected on important potential confounders such as digital literacy, communication style, level of dental anxiety, or socioeconomic background, all of which may influence perceptions of empathy and communication quality. Third, the cross-sectional design does not allow causal inferences or conclusions about temporal changes in communication behavior. Fourth, the evaluation was based solely on written text responses, whereas real clinical communication involves additional non-verbal and paraverbal cues that could meaningfully influence patient perception. Future studies with larger and more diverse samples, inclusion of relevant confounder variables, and multimodal communication formats are needed to validate and extend these findings.

In key areas of communication, particularly in terms of clarity, comprehensibility, and emotional tone, the chatbot outperformed the dentists, as evaluated by both expert reviewers and patients. However, it is important to emphasize that digital systems should not replace direct dentist-patient communication. Rather, artificial intelligence should be viewed as a complementary instrument that enhances, but does not substitute, human interaction. Successful integration of such systems into dental practice will require careful ethical, legal, and practical considerations, as well as broad acceptance among dental professionals and patients alike.

Future studies should include larger and more diverse dentist samples, use multiple respondents per experience level, and apply mixed-effects models to account for clustering within raters. Expanding the design to multimodal communication (e.g., voice, video), multilingual evaluations, and longitudinal assessments would allow for a more comprehensive analysis of AI-supported patient communication. Direct comparisons between different AI systems and human–AI hybrid communication approaches may further clarify the practical integration of such technologies into dental practice.

## 5. Conclusions

The results of our study indicate that ChatGPT was able to generate responses to common dental patient concerns that were not only professionally accurate but also perceived as more empathetic than those of the dentists. These findings suggest that ChatGPT could serve as a valuable support tool in dental communication, especially for preparing and following up on patient interactions and for addressing routine, text-based inquiries in a clear and supportive manner. However, these results must be interpreted with caution due to the exploratory study design, the small and non-representative sample, and the reliance on written, text-only responses that do not capture non-verbal elements of communication. ChatGPT’s perceived empathy reflects simulated language patterns rather than genuine emotional understanding, and the system may still produce inaccurate or inconsistent information. Accordingly, AI-based tools should be viewed as complementary instruments rather than substitutes for direct dentist–patient interaction. Their safe and meaningful integration into dental practice will require careful consideration of ethical, legal, and practical implications, as well as ongoing evaluation as the underlying technology continues to evolve.

## Figures and Tables

**Table 1:** Characteristics of the participating dentists (n = 30).

Variable	Value
Gender distribution	53.33% female (n = 16) 46.67% male (n = 14)
Age	Mean: 28.2 years (SD = 3.06) Range: 24–37 years
Professional experience	3 years: 26.67% (n = 8)
	1 year: 23.33% (n = 7)
	4 years: 20.00% (n = 6)
	2 years: 13.33% (n = 4)
	5 years: 6.67% (n = 2)
	0 years: 3.33% (n = 1)
	8 years: 3.33% (n = 1)
	11 years: 3.33% (n = 1)

This table summarizes the demographic and professional characteristics of the 30 participating dentists, including gender, age, and distribution of years of professional experience.

**Table 2:** Quality ratings of answers from dentists with different experience levels and from ChatGPT.

Question	Group	Response Quality (Mean ± SD)
#1 (How can I effectively protect my teeth from tooth decay?)	<2 Years	2.70 ± 0.54
	2–5 Years	2.33 ± 0.61
	>5 Years	1.03 ± 0.18
	ChatGPT	3.93 ± 0.25
#2 (What causes bad breath, and how can I combat it?)	<2 Years	2.80 ± 0.81
	2–5 Years	1.63 ± 0.67
	>5 Years	1.83 ± 0.91
	ChatGPT	3.73 ± 0.58
#3 (I have had a sharp pain in my upper jaw for a week. What could be the cause, and what should I do now?)	<2 Years	2.27 ± 0.94
	2–5 Years	2.17 ± 0.99
	>5 Years	2.10 ± 1.09
	ChatGPT	3.47 ± 0.90
#4 (My gums bleed when I brush my teeth. What should I do?)	<2 Years	1.87 ± 1.04
	2–5 Years	2.70 ± 0.88
	>5 Years	1.93 ± 0.91
	ChatGPT	3.50 ± 0.82
#5 (What is root canal treatment?)	<2 Years	2.93 ± 0.79
	2–5 Years	2.00 ± 0.64
	>5 Years	1.37 ± 0.72
	ChatGPT	3.70 ± 0.60
#6 (Does smoking affect oral health?)	<2 Years	2.03 ± 0.93
	2–5 Years	2.27 ± 0.74
	>5 Years	2.53 ± 1.33
	ChatGPT	3.13 ± 1.14
#7 (My tooth is pulsating after a filling. What should I do?)	<2 Years	1.70 ± 0.88
	2–5 Years	2.47 ± 0.86
	>5 Years	2.80 ± 1.19
	ChatGPT	3.03 ± 1.10
#8 (How does my diabetes mellitus affect my dental health?)	<2 Years	2.73 ± 0.83
	2–5 Years	2.43 ± 0.90
	>5 Years	1.40 ± 0.72
	ChatGPT	3.43 ± 0.97
#9 (I am afraid of dental treatment. What can I expect during a filling procedure?)	<2 Years	1.67 ± 0.66
	2–5 Years	2.83 ± 0.70
	>5 Years	1.80 ± 1.03
	ChatGPT	3.67 ± 0.61
#10 (I have brown spots in my fissures. Is this tooth decay?)	<2 Years	2.57 ± 0.82
	2–5 Years	2.93 ± 0.91
	>5 Years	1.07 ± 0.25
	ChatGPT	3.43 ± 0.63

Answer quality to 10 standardized dental questions was evaluated by 30 independent dentists using a 4-point rating scale (1 = low quality, 4 = high quality). Shown are mean values ± standard deviations for the three dentist groups (<2, 2–5, >5 years) and ChatGPT.

**Table 3:** Pairwise comparison of answer quality across dentist experience groups and ChatGPT.

	Comparison		
Question	Group	Group	*p*-Value
#1 (How can I effectively protect my teeth from tooth decay?)	ChatGPT	<2 Years	0.0012
	ChatGPT	2–5 Years	*p* < 0.0001
	ChatGPT	>5 Years	*p* < 0.0001
	>5 Years	2–5 Years	0.0005
	>5 Years	<2 Years	*p* < 0.0001
	2–5 Years	<2 Years	1.0000
#2 (What causes bad breath, and how can I combat it?)	ChatGPT	<2 Years	0.0332
	ChatGPT	2–5 Years	*p* < 0.0001
	ChatGPT	>5 Years	*p* < 0.0001
	>5 Years	2–5 Years	1.0000
	>5 Years	<2 Years	0.0240
	2–5 Years	<2 Years	0.0027
#3 (I have had a sharp pain in my upper jaw for a week. What could be the cause, and what should I do now?)	ChatGPT	<2 Years	0.0018
	ChatGPT	2–5 Years	0.0005
	ChatGPT	>5 Years	0.0002
	>5 Years	2–5 Years	1.0000
	>5 Years	<2 Years	1.0000
	2–5 Years	<2 Years	1.0000
#4 (My gums bleed when I brush my teeth. What should I do?)	ChatGPT	<2 Years	*p* < 0.0001
	ChatGPT	2–5 Years	0.1091
	ChatGPT	>5 Years	*p* < 0.0001
	>5 Years	2–5 Years	0.1432
	>5 Years	<2 Years	1.0000
	2–5 Years	<2 Years	0.0823
#5 (What is root canal treatment?)	ChatGPT	<2 Years	0.1432
	ChatGPT	2–5 Years	*p* < 0.0001
	ChatGPT	>5 Years	*p* < 0.0001
	>5 Years	2–5 Years	0.3840
	>5 Years	<2 Years	*p* < 0.0001
	2–5 Years	<2 Years	0.0332
#6 (Does smoking affect oral health?)	ChatGPT	<2 Years	0.0058
	ChatGPT	2–5 Years	0.0454
	ChatGPT	>5 Years	0.3840
	>5 Years	2–5 Years	1.0000
	>5 Years	<2 Years	0.8852
	2–5 Years	<2 Years	1.0000
#7 (My tooth is pulsating after a filling. What should I do?)	ChatGPT	<2 Years	0.0003
	ChatGPT	2–5 Years	0.5938
	ChatGPT	>5 Years	1.0000
	>5 Years	2–5 Years	1.0000
	>5 Years	<2 Years	0.0058
	2–5 Years	<2 Years	0.1432
#8 (How does my diabetes mellitus affect my dental health?)	ChatGPT	<2 Years	0.2391
	ChatGPT	2–5 Years	0.0171
	ChatGPT	>5 Years	*p* < 0.0001
	>5 Years	2–5 Years	0.0121
	>5 Years	<2 Years	0.0003
	2–5 Years	<2 Years	1.0000
#9 (I am afraid of dental treatment. What can I expect during a filling procedure?)	ChatGPT	<2 Years	*p* < 0.0001
	ChatGPT	2–5 Years	0.0823
	ChatGPT	>5 Years	*p* < 0.0001
	>5 Years	2–5 Years	0.0085
	>5 Years	<2 Years	1.0000
	2–5 Years	<2 Years	0.0018
#10 (I have brown spots in my fissures. Is this tooth decay?)	ChatGPT	<2 Years	0.0615
	ChatGPT	2–5 Years	0.8852
	ChatGPT	>5 Years	*p* < 0.0001
	>5 Years	2–5 Years	*p* < 0.0001
	>5 Years	<2 Years	*p* < 0.0001
	2–5 Years	<2 Years	1.0000

This table shows the results of the pairwise statistical comparisons of response quality between the three dentist experience groups (<2, 2–5, >5 years) and ChatGPT across 10 standardized dental questions. Significance levels from the exact post hoc tests following the Friedman test are reported.

**Table 4:** Characteristics of the patient sample (n = 50).

Variable	Value
Gender distribution	62% female (n = 31) 38% male (n = 19)
Age	Mean: 47.7 years (SD = 19.1) Range: 21–80 years

This table summarizes the demographic characteristics of the 50 participating patients, including gender distribution and age.

**Table 5:** Summary of empathy ratings (mean ± SD) across groups.

Group	Mean ± SD
<2 Years	2.52 ± 0.89
2–5 Years	2.42 ± 0.89
>5 Years	1.58 ± 0.89
ChatGPT	3.27 ± 0.95

This table summarizes the average empathy ratings provided by 50 patients for the responses of the three dentist experience groups (<2 years, 2–5 years, >5 years) and ChatGPT across all 10 questions. Ratings were given on a 4-point scale (1 = low empathy, 4 = high empathy). Mean values and average standard deviations are reported.

**Table 6:** Empathy ratings of answers from dentists and ChatGPT.

Question	Group	Response Quality (Mean ± SD)
#1 (How can I effectively protect my teeth from tooth decay?)	<2 Years	3.06 ± 0.87
	2–5 Years	2.26 ± 0.80
	>5 Years	1.46 ± 0.95
	ChatGPT	3.04 ± 1.05
#2 (What causes bad breath, and how can I combat it?)	<2 Years	3.04 ± 1.03
	2–5 Years	2.22 ± 0.95
	>5 Years	1.68 ± 0.98
	ChatGPT	2.88 ± 1.06
#3 (I have had a sharp pain in my upper jaw for a week. What could be the cause, and what should I do now?)	<2 Years	2.78 ± 0.93
	2–5 Years	2.60 ± 0.86
	>5 Years	1.28 ± 0.73
	ChatGPT	3.28 ± 0.93
#4 (My gums bleed when I brush my teeth. What should I do?)	<2 Years	1.82 ± 0.94
	2–5 Years	3.10 ± 1.02
	>5 Years	1.88 ± 0.82
	ChatGPT	3.10 ± 1.02
#5 (What is root canal treatment?)	<2 Years	2.62 ± 1.01
	2–5 Years	2.06 ± 0.87
	>5 Years	1.76 ± 0.98
	ChatGPT	3.44 ± 0.81
#6 (Does smoking affect oral health?)	<2 Years	2.52 ± 0.91
	2–5 Years	2.70 ± 0.68
	>5 Years	1.24 ± 0.74
	ChatGPT	3.40 ± 0.93
#7 (My tooth is pulsating after a filling. What should I do?)	<2 Years	1.64 ± 0.94
	2–5 Years	2.60 ± 0.93
	>5 Years	2.62 ± 1.12
	ChatGPT	3.02 ± 1.06
#8 (How does my diabetes mellitus affect my dental health?)	<2 Years	2.58 ± 0.97
	2–5 Years	2.52 ± 0.95
	>5 Years	1.44 ± 0.76
	ChatGPT	3.34 ± 0.96
#9 (I am afraid of dental treatment. What can I expect during a filling procedure?)	<2 Years	1.38 ± 0.60
	2–5 Years	2.54 ± 1.05
	>5 Years	2.64 ± 1.01
	ChatGPT	3.40 ± 0.83
#10 (I have brown spots in my fissures. Is this tooth decay?)	<2 Years	2.78 ± 0.84
	2–5 Years	2.54 ± 0.84
	>5 Years	1.30 ± 0.79
	ChatGPT	3.36 ± 0.88

This table presents the empathy ratings assigned by 50 patients to the responses of the three dentist experience groups (<2, 2–5, >5 years) and ChatGPT across 10 standardized dental questions. Each response was evaluated on a 4-point scale (1 = low empathy, 4 = high empathy). Mean values ± standard deviations are reported.

**Table 7:** Pairwise comparison of empathy ratings for dentists and ChatGPT.

	Comparison		
Question	Group	Group	*p*-Value
#1 (How can I effectively protect my teeth from tooth decay?)	ChatGPT	<2 Years	1.0000
	ChatGPT	2–5 Years	0.0161
	ChatGPT	>5 Years	*p* < 0.0001
	>5 Years	2–5 Years	0.0094
	>5 Years	<2 Years	*p* < 0.0001
	2–5 Years	<2 Years	0.0123
#2 (What causes bad breath, and how can I combat it?)	ChatGPT	<2 Years	1.0000
	ChatGPT	2–5 Years	0.0690
	ChatGPT	>5 Years	*p* < 0.0001
	>5 Years	2–5 Years	0.2388
	>5 Years	<2 Years	*p* < 0.0001
	2–5 Years	<2 Years	0.0094
#3 (I have had a sharp pain in my upper jaw for a week. What could be the cause, and what should I do now?)	ChatGPT	<2 Years	0.4113
	ChatGPT	2–5 Years	0.0690
	ChatGPT	>5 Years	*p* < 0.0001
	>5 Years	2–5 Years	*p* < 0.0001
	>5 Years	<2 Years	*p* < 0.0001
	2–5 Years	<2 Years	1.0000
#4 (My gums bleed when I brush my teeth. What should I do?)	ChatGPT	<2 Years	*p* < 0.0001
	ChatGPT	2–5 Years	1.0000
	ChatGPT	>5 Years	*p* < 0.0001
	>5 Years	2–5 Years	*p* < 0.0001
	>5 Years	<2 Years	1.0000
	2–5 Years	<2 Years	*p* < 0.0001
#5 (What is root canal treatment?)	ChatGPT	<2 Years	0.0123
	ChatGPT	2–5 Years	*p* < 0.0001
	ChatGPT	>5 Years	*p* < 0.0001
	>5 Years	2–5 Years	1.0000
	>5 Years	<2 Years	0.0030
	2–5 Years	<2 Years	0.1317
#6 (Does smoking affect oral health?)	ChatGPT	<2 Years	0.0054
	ChatGPT	2–5 Years	0.0550
	ChatGPT	>5 Years	*p* < 0.0001
	>5 Years	2–5 Years	*p* < 0.0001
	>5 Years	<2 Years	*p* < 0.0001
	2–5 Years	<2 Years	1.0000
#7 (My tooth is pulsating after a filling. What should I do?)	ChatGPT	<2 Years	*p* < 0.0001
	ChatGPT	2–5 Years	0.7867
	ChatGPT	>5 Years	0.7867
	>5 Years	2–5 Years	1.0000
	>5 Years	<2 Years	0.0009
	2–5 Years	<2 Years	0.0009
#8 (How does my diabetes mellitus affect my dental health?)	ChatGPT	<2 Years	0.0268
	ChatGPT	2–5 Years	0.0161
	ChatGPT	>5 Years	*p* < 0.0001
	>5 Years	2–5 Years	0.0002
	>5 Years	<2 Years	0.0001
	2–5 Years	<2 Years	1.0000
#9 (I am afraid of dental treatment. What can I expect during a filling procedure?)	ChatGPT	<2 Years	*p* < 0.0001
	ChatGPT	2–5 Years	0.0054
	ChatGPT	>5 Years	0.0208
	>5 Years	2–5 Years	1.0000
	>5 Years	<2 Years	*p* < 0.0001
	2–5 Years	<2 Years	*p* < 0.0001
#10 (I have brown spots in my fissures. Is this tooth decay?)	ChatGPT	<2 Years	0.1970
	ChatGPT	2–5 Years	0.0094
	ChatGPT	>5 Years	*p* < 0.0001
	>5 Years	2–5 Years	*p* < 0.0001
	>5 Years	<2 Years	*p* < 0.0001
	2–5 Years	<2 Years	1.0000

This table shows the results of the pairwise statistical comparisons of empathy ratings provided by 50 patients for the three dentist experience groups (<2, 2–5, >5 years) and ChatGPT across 10 standardized dental questions. Significance levels from the exact post hoc tests following the Friedman test are reported.

## Data Availability

The datasets generated and analyzed during the current study are not publicly available due to privacy and institutional data protection regulations but are available from the corresponding author upon reasonable request.

## Supplementary Materials

The following supporting information can be downloaded at: https://www.mdpi.com/article/10.3390/healthcare13233099/s1, Figure S1: Mean quality ratings (±95% CI) for the answers provided by the three dentist groups (<2 years, 2–5 years, >5 years of professional experience) and ChatGPT across all 10 questions. Figure S2: Comparison of mean quality ratings (±95% CI) for the answers provided by the three dentist groups (<2 years, 2–5 years, >5 years of professional experience) and ChatGPT across all 10 questions. Significant differences between groups are indicated by small squares above the respective bars. Figure S3: Mean empathy ratings (±95% CI) provided by 50 patients for the responses of the three dentist groups (<2 years, 2–5 years, >5 years of professional experience) and ChatGPT across all 10 questions. Higher values indicate higher perceived empathy. Figure S4: Comparison of mean empathy ratings (±95% CI) provided by 50 patients for the three dentist groups (<2 years, 2–5 years, >5 years of professional experience) and ChatGPT across all 10 questions. Significant differences between groups are indicated by small squares above the corresponding bars. Figure S5: Correlation between the Evaluation of response quality and empathy.
