# Supplementary material for: Professional Quality and Empathy of Responses Provided by AI and Dentists: A Cross-Sectional Study
Source: Healthcare (Basel). 2025 Nov 27;13(23):3099. doi: 10.3390/healthcare13233099 (PMC12691705; doi:10.3390/healthcare13233099)
Supplement: Supplementary file 1 [file healthcare-13-03099-s001.zip › healthcare-3964127-supplementary.pdf]

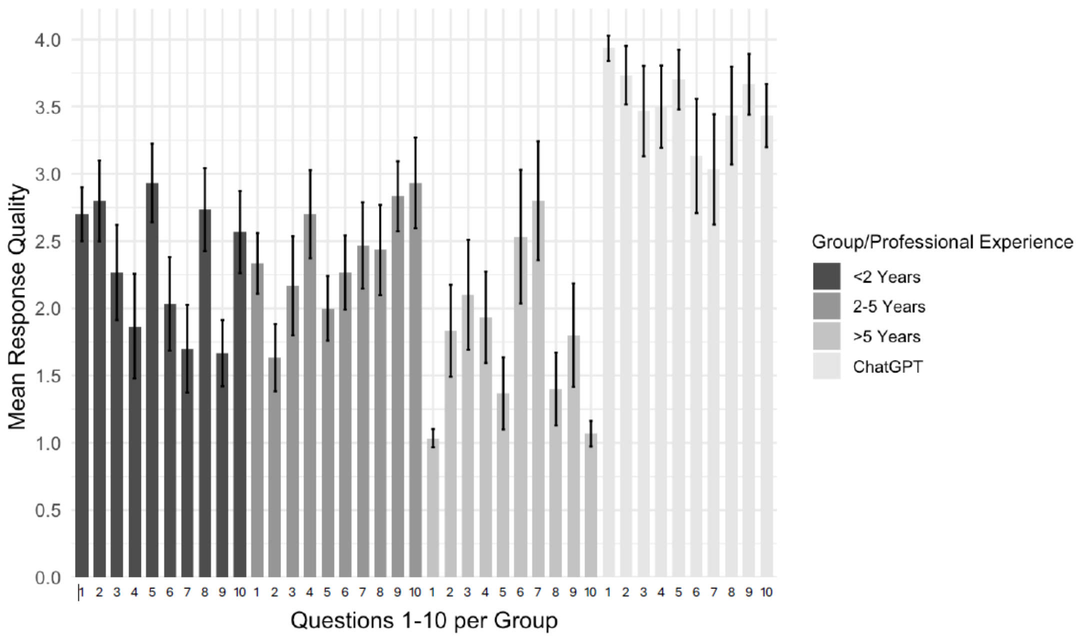

Figure S1: Mean quality ratings (±95% CI) for the answers provided by the three dentist groups (<2 years, 2–5 years, >5 years of professional experience) and ChatGPT across all 10 questions.

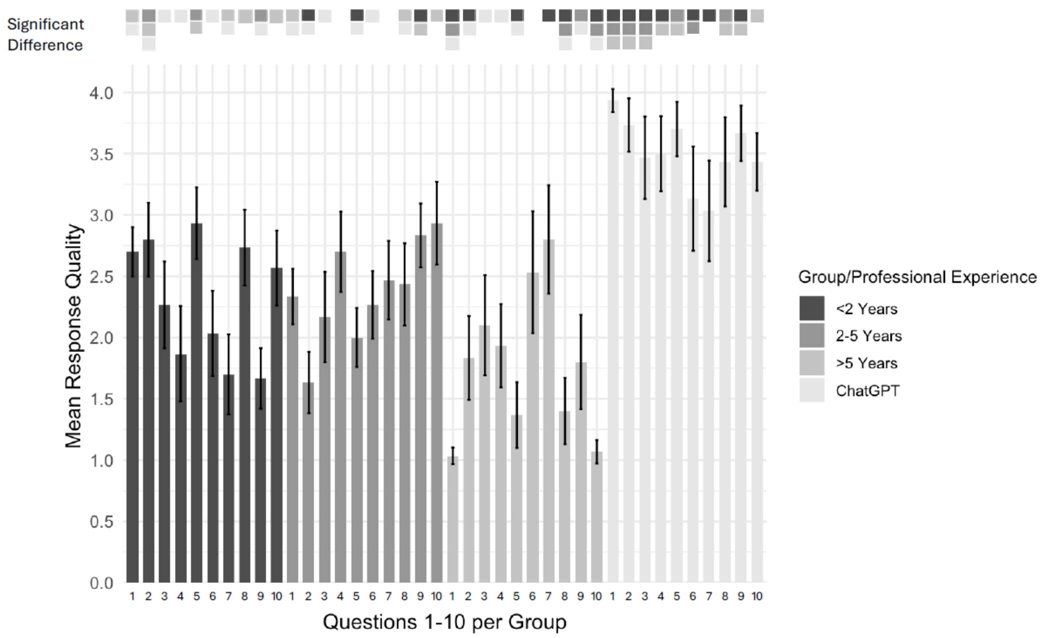

Figure S2: Comparison of mean quality ratings (±95% CI) for the answers provided by the three dentist groups (<2 years, 2–5 years, >5 years of professional experience) and ChatGPT across all 10 questions. Significant differences between groups are indicated by small squares above the respective bars.

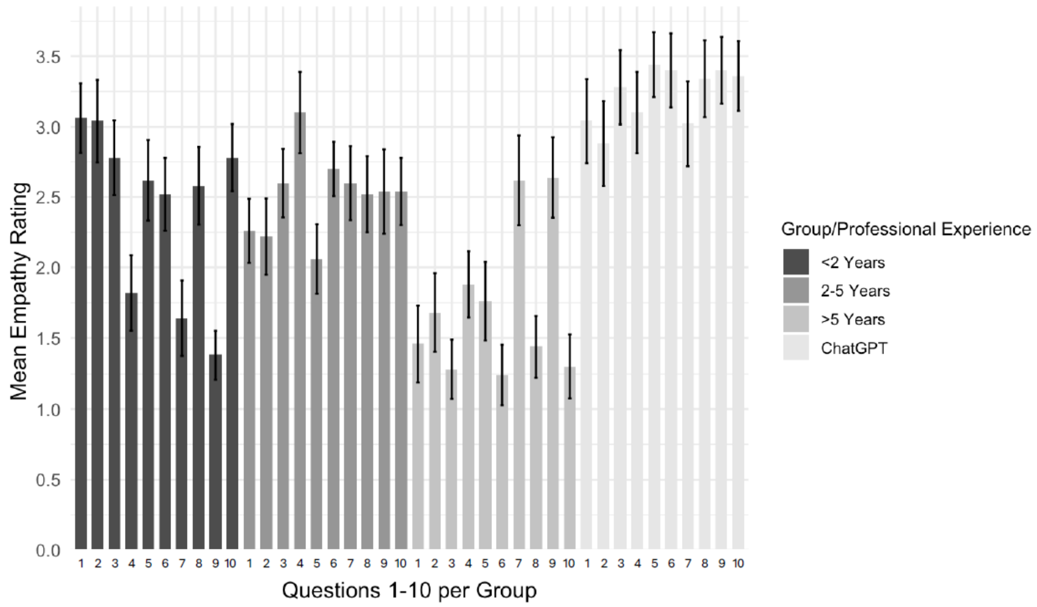

Figure S3: Mean empathy ratings (±95% CI) provided by 50 patients for the responses of the three dentist groups (<2 years, 2–5 years, >5 years of professional experience) and ChatGPT across all 10 questions. Higher values indicate higher perceived empathy.

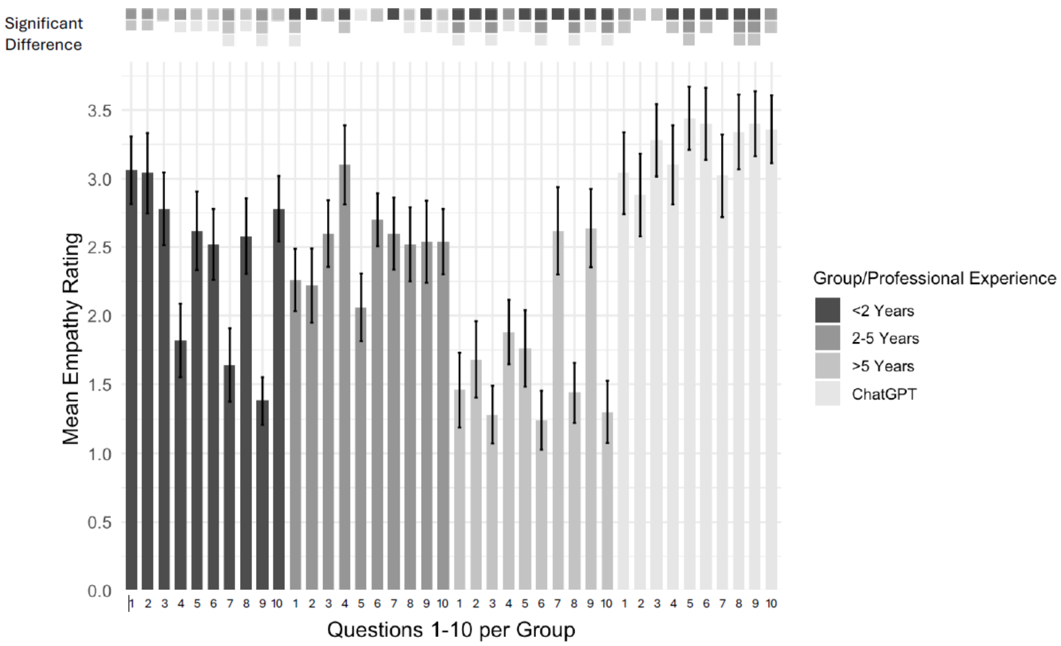

Figure S4: Comparison of mean empathy ratings (±95% CI) provided by 50 patients for the three dentist groups (<2 years, 2–5 years, >5 years of professional experience) and ChatGPT across all 10 questions. Significant differences between groups are indicated by small squares above the corresponding bars.

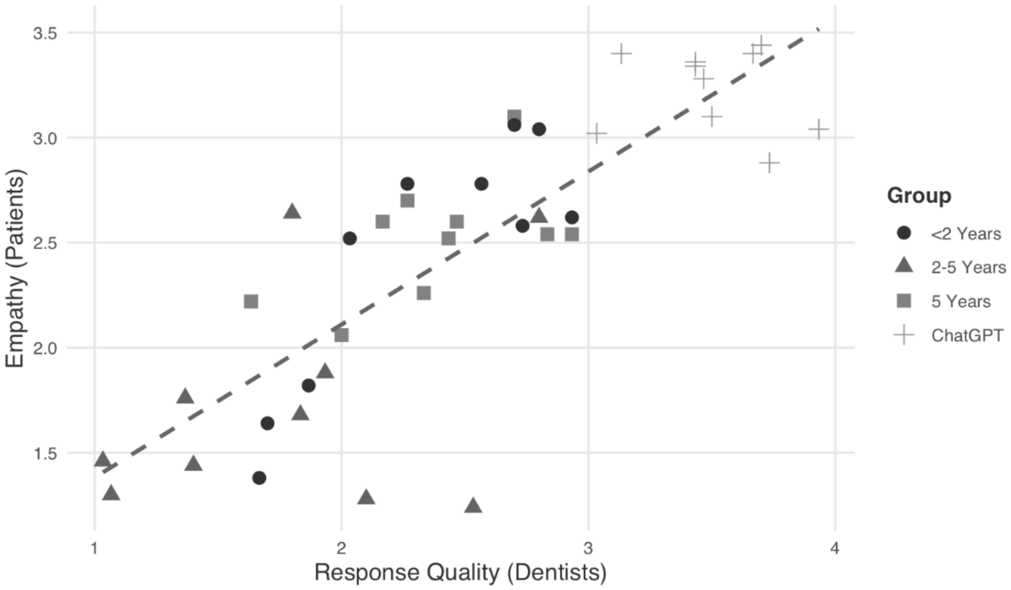

Figure S5: Correlation between the Evaluation of response quality and empathy
